# *SiASR4*, the Target Gene of SiARDP from *Setaria italica*, Improves Abiotic Stress Adaption in Plants

**DOI:** 10.3389/fpls.2016.02053

**Published:** 2017-01-12

**Authors:** Jianrui Li, Yang Dong, Cong Li, Yanlin Pan, Jingjuan Yu

**Affiliations:** State Key Laboratory of Agrobiotechnology, College of Biological Sciences, China Agricultural UniversityBeijing, China

**Keywords:** *Setaria italica*, foxtail millet, *SiASR4*, salt, drought, ABA-dependent pathway

## Abstract

Drought and other types of abiotic stresses negatively affect plant growth and crop yields. The abscisic acid-, stress-, and ripening-induced (ASR) proteins play important roles in the protection of plants against abiotic stress. However, the regulatory pathway of the gene encoding this protein remains to be elucidated. In this study, the foxtail millet (*Setaria italica*) ASR gene, *SiASR4*, was cloned and characterized. SiASR4 localized to the cell nucleus, cytoplasm and cytomembrane, and the protein contained 102 amino acids, including an ABA/WDS (abscisic acid/water-deficit stress) domain, with a molecular mass of 11.5 kDa. The abundance of *SiASR4* transcripts increased after treatment with ABA, NaCl, and PEG in foxtail millet seedlings. It has been reported that the *S. italica* ABA-responsive DRE-binding protein (SiARDP) binds to a DNA sequence with a CCGAC core and that there are five dehydration-responsive element (DRE) motifs within the *SiASR4* promoter. Our analyses demonstrated that the SiARDP protein could bind to the *SiASR4* promoter *in vitro* and *in vivo*. The expression of *SiASR4* increased in *SiARDP*-overexpressing plants. *SiASR4*-transgenic *Arabidopsis* and *SiASR4*-overexpressing foxtail millet exhibited enhanced tolerance to drought and salt stress. Furthermore, the transcription of stress-responsive and reactive oxygen species (ROS) scavenger-associated genes was activated in *SiASR4* transgenic plants. Together, these findings show that *SiASR4* functions in the adaption to drought and salt stress and is regulated by *SiARDP* via an ABA-dependent pathway.

## Introduction

Plants are challenged by many severe environmental conditions, among which drought and salinity are two significant abiotic stresses that reduce crop productivity. However, plants can cope with abiotic stress via intricate mechanisms, including morphological, physiological, and molecular responses (Farooq et al., 2009). A large number of proteins have been reported to be involved in abiotic stress in plants, such as molecular chaperones (Wang et al., 2004), osmoregulatory proteins (Tamura et al., 2003), ion channel proteins (Ward et al., 1995), and transcription factors (Baldoni et al., 2015). Therefore, the identification and functional analysis of stress-associated proteins is helpful for plant breeders to cultivate stress-tolerant plants (Fujita et al., 2005).

Abscisic acid-, stress-, and ripening-induced (ASR) proteins, which are types of plant-specific proteins, were first identified in cultivated tomato. They are induced by water deficit and fruit ripening (Iusem et al., 1993). Since then, many ASR homologous proteins have been cloned from a variety of plants (Cakir et al., 2003; Kalifa et al., 2004; Virlouvet et al., 2011; Hu et al., 2013; Zhang et al., 2015). ASR gene families are widely distributed in monocots, dicots, herbs, and xylophyta, but no ASR ortholog have been identified in *Arabidopsis* (Wong et al., 2006). ASRs are heat-stable and highly hydrophilic proteins with low molecular weights (Iusem et al., 1993). All known ASR proteins have been shown to possess a zinc-binding domain at the N-terminal end and a putative nuclear targeting signal at the C-terminal end (Cakir et al., 2003). ASRs display different subcellular localizations. Some of these proteins localize to the nucleus (Padmanabhan et al., 1997), while some are detected in both the cytoplasm and nucleus (Kalifa et al., 2004), and some are dispersed throughout the cell (Wang L. et al., 2016), likely reflecting their different functions.

There are a large number of ASRs reported in response to ABA and abiotic stress. The tomato ASR gene, *SlASR1*, is induced during high-salinity, osmotic and ABA treatments (Konrad and Bar-Zvi, 2008). A wheat ASR gene, *TaASR1*, increases drought and osmotic stress tolerance in transgenic tobacco by activating the antioxidant system and the expression levels of stress-responsive genes (Hu et al., 2013). Overexpression of banana ASR (*MaASR*) in *Arabidopsis* and rice ASRs (*OsASR1* and *OsASR3*) in rice has been shown to enhance drought and salt tolerance, respectively, in transgenic lines (Joo et al., 2013; Zhang et al., 2015). The loblolly pine ASR gene *lp3* is induced by water deficit stress mediated by ABA (Padmanabhan et al., 1997). A *Salicornia brachiata* ASR gene, *SbASR1*, enhances drought and high-salinity tolerance in transgenic groundnut (Tiwari et al., 2015). In foxtail millet, six ASR genes were identified. The expression levels of these genes are increased in response to various stress treatments. Overexpression of *SiASR1* in tobacco enhances drought and oxidative tolerance by regulating oxidative-related genes (Feng et al., 2016). Because ASRs localize to the cytoplasm or nucleus, they may act as molecular chaperones or transcription factors. In tomato, the unstructured form of *SlASR1* in the cytosol can stabilize a number of proteins to prevent protein denaturation caused by repeated freeze-thaw cycles. This finding suggested that SlASR1 exhibits a chaperone-like activity in the cytosol (Konrad and Bar-Zvi, 2008). In wheat, *TaASR1* functioned as a positive factor in the regulation of stress-responsive and reactive oxygen species (ROS)-related gene expression in response to drought and osmotic stress (Hu et al., 2013). *SbASR1* can reduce the accumulation of H_2_O_2_ and ${\text{O}}_{2}^{.-}$ radicals and induce the transcription of ROS scavenger-associated genes (Tiwari et al., 2015). The rice ASR gene *OsASR5* binds to *cis* elements in the promoters of aluminum-responsive genes and regulates the expression of these genes (Arenhart et al., 2016). The transcription levels of some ABA/stress-responsive genes decrease in transgenic plants carrying the lily ASR gene *LLA23*, suggesting that *LLA2*3 acts as a transcription factor mediating stress-responsive ABA signaling (Yang et al., 2005). In addition, transcriptome and proteome analyses showed that ASRs play a prominent role in the plant response to ABA and resistance to abiotic stress (Virlouvet et al., 2011; Ricardi et al., 2014). When ChIP-seq was used to analyze the target genes of tomato ASR1, some drought stress-related and aquaporin genes were identified (Ricardi et al., 2014). Transcriptomic analysis of maize leaves revealed that *ZmASR1, ZmASR2, ZmASR4*, and *ZmASR7-1* are upregulated under water deficit stress and that *ZmASR1, ZmASR3*, and *ZmASR4* are upregulated under ABA treatment (Virlouvet et al., 2011).

Foxtail millet (*Setaria italica*), an important grain crop in China and India, can grow in arid or marginal soils (Barton et al., 2009). Because of its prominent drought tolerance, small diploid genome size (~510 Mb), self-pollination and short life cycle, it is an ideal model system for studying C_4_ crops and biofuel grasses (Zhang et al., 2012; Wang Y. et al., 2016). A reference genome sequence of foxtail millet was recently generated (Bennetzen et al., 2012). Because of the remarkable drought tolerance and water use efficiency of foxtail millet, the identification of stress-relevant genes in this species is important. In the present study, an ASR gene, *SiASR4*, was cloned from foxtail millet cDNA. It was induced by ABA treatment as well as drought and high-salinity treatments. Overexpression of *SiASR4* resulted in enhanced tolerance to abiotic stress in transgenic *Arabidopsis* and foxtail millet. However, there are no differences of the *SiASR4*-RNAi foxtail millet compared with WT plants under stress treatments. *SiARDP* is induced by abiotic stress and ABA treatment. It plays a critical role in response to abiotic stress (Li et al., 2014). SiARDP binds to DRE *cis*-elements in the *SiASR4* promoter region both *in vivo and in vitro*. Moreover, *SiASR4* transcription is increased in *SiARDP*-overexpressing plants. These results showed that *SiASR4* plays an important role in response to salt and drought stress, and may be regulated by *SiARDP* via an ABA-dependent signaling pathway. These findings reveal the potential of the application of *SiASR4* to engineer other crops with improved resistance to drought and salt stress.

## Materials and methods

### Plant materials and growth conditions

Foxtail millet (*S. italica*; Jigu 11) seeds were germinated on moist filter paper for 24 h at 28°C and then planted in a soil mixture (nutrient soil: Vermiculite, 1:1, v/v) in a growth chamber (16-h light/8-h dark, 26–28°C, 60% relative humidity). After 2 weeks, the soil mixture on the seedling roots were washed away, and the seedlings were transferred to 1/4 Hoagland solution for 3 days. They were then subjected to treatment with 100 mM NaCl, 20% PEG6000 or 10 μM ABA and were harvested at the indicated times. Seedlings cultured in 1/4 Hoagland were used as controls. For tissue expression analyses, the leaves, stems and roots of 2-week-old seedlings and mature seeds were harvested. All samples were stored at −80°C after being frozen in liquid nitrogen.

Seeds of *Arabidopsis thaliana* (Col-0) were surface-sterilized and plated on MS medium containing 2% sucrose and 0.8% agar and incubated for 72 h at 4°C before being transferred to 22°C and a 16-h light/8-h dark photoperiod for germination. After 5 days, the seedlings were planted in a soil mixture (nutrient soil: Vermiculite, 1:1, v/v) and grown in the same conditions. *Nicotiana benthamiana* seeds were planted in a potting soil mixture (nutrient soil: Vermiculite, 1:1, v/v) and grown in a growth chamber under a 16-h light/8-h dark photoperiod for germination at 22–23°C.

### RNA extraction and RNA analysis

Total RNA was extracted from foxtail millet and *Arabidopsis* using the TRIzol reagent (Invitrogen, USA). After digestion with DNaseI (Takara, Japan), 3–5 μg of total RNA was prepared, and cDNA was synthesized via reverse transcription using M-MLV Reverse Transcriptase (Promega, USA). Semi-quantitative RT-PCR was performed using 2 × Taq PCR StarMix with Loading Dye (GenStar, China). The PCR conditions were 95°C for 5 min, followed by 25 cycles of 95°C for 30 s, 60°C for 30 s, and 72°C for 30 s, with a final step at 72°C for 10 min. Quantitative RT-PCR (qRT-PCR) was performed using 2 × Ultra SYBR Mixture (CWBIO, China) on a qTower 2.2 Real-Time PCR System (AnalytikJena, Germany). The PCR conditions were 95°C for 10 min, followed by 40 cycles of 95°C for 15 s, and 60°C for 1 min. Relative gene expression levels were calculated using the 2^−ΔΔCT^ method (Livak and Schmittgen, 2001).

### GUS staining

For GUS staining, fresh plant samples were immersed in GUS staining buffer containing a 1 mM 5-bromo-4-chloro-3-indolyl glucuronide (X-Gluc) solution in 100 mM sodium phosphate buffer (pH 7.0) with 0.5 M EDTA, 5 mM FeK_3_(CN)_6_, 5 mM FeK_4_(CN)_6_, and 0.1% Triton X-100. Following vacuum infiltration, the samples were stained at 37°C overnight, and the GUS staining solution was then replaced with 70% ethanol for decolorization. Finally, the samples were photographed under a dissecting microscope (Olympus SEX16, Japan).

### Subcellular localization of the *SiASR4* protein

To generate a GFP-fusion protein, the coding sequence of *SiASR4* without the stop codon was amplified and inserted into the *Bam*HI and *Sma*I sites of the pROK219-GFP plasmid and the *Xba*I/*Kpn*I sites of the pSuper1300-GFP plasmid. The pROK219-GFP and pROK219-SiASR4-GFP plasmids were used for transformation. Foxtail millet seeds were germinated on moist filter paper for 24 h at 28°C and then grown in pots filled with nutrient soil and vermiculite mixed 1:1 (v/v) under 12 h-light/12 h-dark conditions at 28°C for 3 days, after which they were moved to dark conditions for 4–6 days. Stem and leaf tissues were cut into 0.5–1-mm-wide strips. Protoplasts were prepared and transformed according to the procedure described by Zhang et al. (2011). To prepare protoplasts, the tissues were transferred into 0.6 M mannitol and incubated for 10 min to plasmolyse, and then enzyme solution was used to digest tissues in the dark for 4–5 h. The protoplasts were collected through 74 μm nylon meshes. For transformation, 200 μL protoplasts (1 × 10^6^ cells mL^−1^) were mixed with 20 μg plasmid DNA and 220 μL 40% PEG, and the samples were cultured for 16–20 h. Fluorescence was observed using a confocal laser microscope (Leica HQ).

The pSuper1300-GFP and pSuper1300-SiASR4-GFP plasmids were transformed into *Agrobacterium* strain EHA105. The *Agrobacterium* lines were then resuspended in transformation buffer and infiltrated into *N. benthamiana* leaves. These plants were grown at 22–23°C for 3 days, and fluorescence in the leaves was imaged using a confocal laser microscope (Leica HQ).

### Transcriptional activation assay in yeast cells

Transcriptional activation of SiASR4 was performed in the yeast strain YRG-2. The full-length *SiASR4* sequence was inserted into the *Eco*RI and *Sma*I sites of the pBD-GAL4 plasmid (Stratagene, USA), driven by the yeast alcohol dehydrogenase1 (ADH1) promoter. The pBD-GAL4 and pGAL4 plasmid were used as negative and positive controls, respectively. The plasmids were transformed into yeast cells independently. These transformed yeast cells were grown on SD/Trp- or SD/Trp-/His- medium for 3 days at 30°C.

### Generation of *SiASR4* transgenic *Arabidopsis* plants

For *SiASR4* overexpression in *Arabidopsis*, the full-length *SiASR4* sequence without the stop codon fused to a flag tag was amplified via RT-PCR and cloned into the modified binary vector pCAMBIA2300 at the *Kpn*I and *Sac*I sites, controlled by the CaMV 35S promoter. For the *SiASR4* promoter assay, the putative *SiASR4* promoter was amplified and inserted into the pCAMBIA1391 vector at the *Hin*dIII and *Eco*RI sites to drive the GUS gene. The plasmids were transformed into *Agrobacterium* strain GV3101. Infection of *Arabidopsis* Col-0 was performed using the floral dip method (Zhang et al., 2006). Developing floral tissues were dipped into *Agrobacterium* solution containing 5% sucrose and Silwet-77 (500 μL L^−1^). After inoculation, the plants were placed under a plastic dome for 12–24 h and then grown under a 16-h light/8-h dark photoperiod at 22°C. Seeds were obtained following self-pollination. The *SiASR4* and *proSiASR4* transgenic *Arabidopsis* seedlings were selected on MS medium containing kanamycin (50 μg/mL) or hygromycin (15 μg/mL) respectively and then confirmed by PCR. The expression of *SiASR4* in transgenic *Arabidopsis* was determined by semi-qRT-PCR using *SiASR4* (qRT)-F/R primer pairs (Table S1). Three independent homozygous T3 lines with kanamycin resistance and high expression levels of *SiASR4* were chosen for further studies.

### Transformation and regeneration of *SiASR4* transgenic foxtail millet

To construct the *SiASR4*-overexpressing vector, the coding region fused to a flag tag at the C-terminus was amplified and inserted into the *Bam*HI and *Sac*I sites of the modified binary vector pCOU under the control of the ubiquitin promoter. To generate *SiASR4*-RNAi constructs, the *SiASR4* sequence with *Kpn*I and *Sac*I sites was amplified and inserted between the GUS gene and the NOS terminator, and the *SiASR4* sequence with *Spe*I and *Bam*HI sites was amplified and inserted in reverse orientation between the ubiquitin promoter and GUS gene of the modified binary vector pCOU. The *SiASR4*-overexpressing and *SiASR4*-RNAi constructs were first introduced into *Agrobacterium tumefaciens* strain LBA4404. The transformation of foxtail millet was performed according to a previously described method (Wang et al., 2011). 0.5–1-cm-long immature inflorescences of the greenhouse-grown Jigu 11 foxtail millet were used as explants. They were cultured on callus induction medium for 30 days to induce embryogenic calli. For transformation, the embryogenic calli were soaked in *Agrobacterium* infection medium (OD_600_ ≈ 0.4–0.6) and inoculated for 20 min. Co-cultivation was performed in the dark at 22°C for 3 days on co-cultivation medium. After co-cultivation, the explants were incubated in the dark at 26°C on resting medium (150 mg/L timentin) for recovery. After 7 days, calli were cultured on selection medium (5 mg/L hygromycin and 150 mg/L timentin) for 2 weeks and then subcultured on selection medium (10 mg/L hygromycin and 150 mg/L timentin) for further 2 weeks, after which they were transferred to regeneration medium (150 mg/L timentin) under a 16-h light/8-h dark condition at 26°C and subcultured every 2 weeks. The regenerated plants were then rooted on rooting medium and further grown in the greenhouse. The *SiASR4*-overexpressing and *SiASR4*-RNAi foxtail millet were determined by PCR using a primer pairs of *SiASR4*-OE-F/R or *SiASR4*-RNAi-F/R, respectively (Table S1). The expression of *SiASR4* in transgenic foxtail millet was examined by qRT-PCR using *SiASR4* (qRT)-F/R primer pairs (Table S1). Two independent lines of T2 generation were chosen for further studies.

### Stress treatment of transgenic *Arabidopsis*

For the germination-stage salt stress analysis, Col-0 and *SiASR4* transgenic *Arabidopsis* seeds were sown on MS medium containing 0, 75, or 100 mM NaCl for 10 days in a growth chamber under 16 h-light/8 h-dark conditions at 22°C. Subsequently, they were photographed, and the fresh weights of Col-0 and transgenic *Arabidopsis* were measured. For the early growth assay under salt stress, Col-0 and transgenic *Arabidopsis* were grown vertically on MS medium for 5 days in a growth chamber under 16 h-light/8 h-dark conditions at 22°C and then transferred to MS medium containing 0, 150, or 200 mM NaCl for 7 days. The seedlings were then subjected to measurement of chlorophyll contents, and the number of lateral roots was counted, after which the plants were photographed. For salt stress in soil, 5-day-old Col-0 and transgenic *Arabidopsis* seedlings grown on MS medium were transferred to soil for an additional 2 weeks. Subsequently, the soil was irrigated with water supplemented with 400 mM NaCl every 7 days for 14 days, and survival rates and chlorophyll contents were analyzed.

For ABA treatment during the germination stage, Col-0 and transgenic *Arabidopsis* seeds were sown on MS medium containing 0, 0.5, or 1 μM ABA for 10 days in a growth chamber under 16 h-light/8 h-dark conditions at 22°C. For the early growth assay, Col-0 and transgenic *Arabidopsis* were grown vertically on MS medium for 5 days at 22°C and then transferred to MS medium containing 0 and 30 μM ABA for 7 days. The resultant phenotypes were then observed.

For dehydration treatment, Col-0 and transgenic *Arabidopsis* seeds were sown on MS medium containing 0, 100, or 200 mM mannitol for 10 days in a growth chamber under 16 h-light/8 h-dark conditions at 22°C. For the early growth assay, Col-0 and transgenic *Arabidopsis* were grown vertically on MS medium for 5 days at 22°C and then transferred to MS medium containing 0 and 300 mM mannitol for 7 days, after which their phenotypes were observed. For the growth assay in soil, 5-day-old Col-0 and transgenic *Arabidopsis* seedlings were grown on MS medium and then transferred to soil for an additional 2 weeks. Irrigation was stopped for 18 days and then resumed for 5 days. Survival rates were calculated, and the chlorophyll content was measured.

### Stress treatment of transgenic foxtail millet

For the germination-stage analysis, seeds of WT, *SiASR4*-overexpressing and *SiASR4*-RNAi transgenic foxtail millet were surface-sterilized and germinated on filter paper containing H_2_O, 100 mM NaCl, 5 μM ABA, and 15% PEG in a growth chamber under 16 h-light/8 h-dark conditions at 28°C. The number of germinated seeds was subsequently counted at 2, 4, 6, and 8 days, and shoot and root lengths were measured on the 6th day. The germination stress index was determined using the following formula: Germination stress index = (germination index under stress treatment/germination index under control treatment) × 100; germination index = [(d2+0.75d4+0.5d6+0.25d8)/total seed number] × 100 (where d2, d4, d6, and d8 represent the number of germinated seeds at 2, 4, 6, and 8 days, respectively).

For the drought stress treatment in soil, 3-week-old WT, *SiASR4*-overexpressing and *SiASR4*-RNAi transgenic foxtail millet seedlings were deprived of water for 13 days, and then watering was resumed for 5 days. For the salt stress treatment in soil, 3-week-old WT, *SiASR4*-overexpressing and *SiASR4*-RNAi foxtail millet seedlings were irrigated with water supplemented with 400 mM NaCl every 7 days for 20 days. Survival rates were calculated, and the chlorophyll content was measured.

### Analysis of chlorophyll contents

The fresh weights of WT and transgenic plant seedlings were determined, and the seedlings were placed in 1.5-mL Eppendorf tubes. Total chlorophyll was extracted with 80% acetone through incubation in the dark for 12 h at room temperature. The samples were then centrifuged at 12,000 rpm for 10 min, and the absorbance was measured at 663 and 645 nm using a microplate reader (BioTek, USA). The chlorophyll content was calculated according to Li et al. (2015): Chlorophyll a = 12.7A663−2.69A645; chlorophyll b = 22.9A645−4.68A663; total chlorophyll = chlorophyll a + chlorophyll b.

### *SiASR4*-regulated gene expression analysis

Total RNA from Col-0 *Arabidopsis* and *SiASR4* transgenic *Arabidopsis* was used to analyze the expression levels of *AtSOD1* (AT1G08830), *AtCAT3* (AT1G20620), *AtSOS1* (AT2G01980), *AtLTP3* (AT5G59320), and *Rd29B* (AT5G52300) via qRT-PCR. Total RNA from Jigu 11 foxtail millet and *SiASR4*-overexpressing foxtail millet was used to analyze the expression levels of *SiSOD* (Seita.9G403600), *SiCAT* (Seita.4G286700), *SiSOS1* (Seita.3G409000), and *SiLTP* (Seita.8G013500) through qRT-PCR. The primers employed for these assays are shown in Table S1.

### Histochemical staining for H_2_O_2_

Three-week-old Col-0 and transgenic *Arabidopsis* grown in pots were not watered and were subjected to salt treatment with 400 mM NaCl for 6 days, respectively, after which the fourth and fifth rosette leaves were harvested. H_2_O_2_ accumulation was detected with diaminobenzidine tetrahydrochloride (DAB). The leaf samples were immersed in DAB solution (1 mg ml^−1^ in water, pH 3.8) and incubated for 4–5 h in the dark with gentle shaking. Following staining, the DAB solution was replaced with bleaching solution (ethanol: Acetic acid: Glycerol = 3:1:1), and the samples were boiled in water at 95°C for 15 min.

### Yeast one-hybrid assay

The bait sequence and mutant bait sequence were synthesized and cloned into the yeast vector pAbAi at the *Hin*dIII and *Xho*I sites (Table S1). According to the protocol of the Yeastmaker™ Yeast Transformation SystemII (Clontech, USA), the bait vectors were linearized through *Bst*BI digestion and transformed into the yeast strain Y1HGold. Positive clones were screened on SD/Ura- medium containing different concentrations of Aureobasidin A (AbA). The *SiARDP* gene was inserted into the *Nde*I and *Xho*I sites of the pGADT7-AD vector according to Li et al. (2014). The resulting plasmid was then transformed into the bait and mutant bait strains and grown on SD/Leu- medium containing 800 ng/ml AbA at 30°C for 3 days.

### Electrophoretic mobility shift assay (EMSA)

*SiARDP* was cloned into the pGEX-TEV vector which contained a GST tag, by Li et al. (2014). This fusion protein was expressed in *E. coli* BL21 cells and purified using Glutathione Sepharose 4B (GE, USA). Oligonucleotides and their reverse complementary oligonucleotides were synthesized. These sequences are shown in **Figure 10**. The probe sequence was labeled with 5′ biotin. Double-stranded probe, competitor probes and mutant probes were obtained through annealing. The reaction mixtures were incubated at room temperature for 30 min and then analyzed via polyacrylamide gel electrophoresis. The electrophoretic mobility shift assay (EMSA) was performed using the LightShift® Chemiluminescent EMSA kit (Thermo, USA).

## Results

### Isolation and sequence analysis of full-length *SiASR4* cDNA

**Figure 1:**
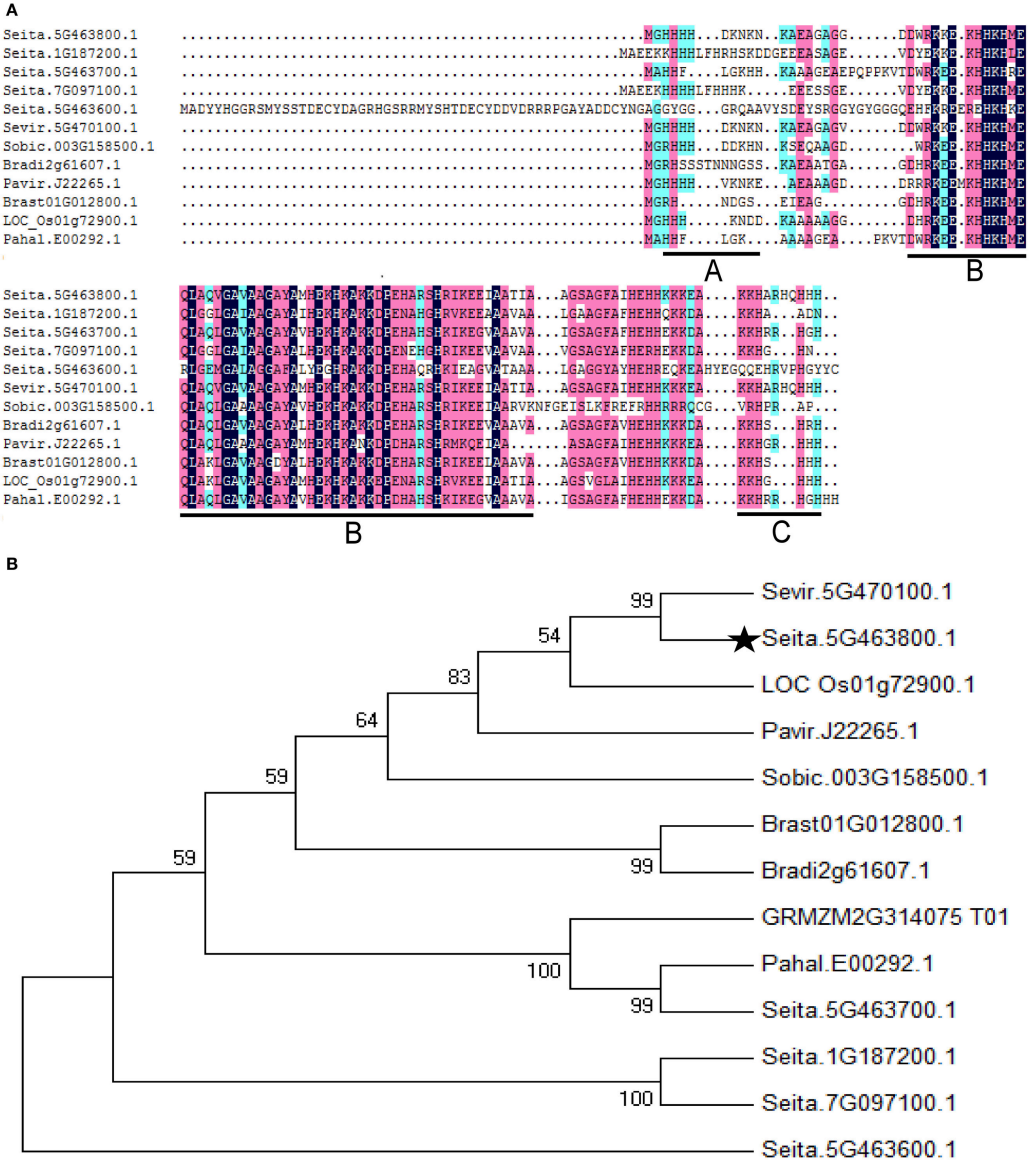
**Multiple amino acid sequence alignment and phylogenetic relationships of the SiASR4 protein and its homologs in grass. (A)** Multiple amino acid sequence alignment of SiASR4 with ASRs from grass species. The zinc-binding region is underlined in area (A); the ABA/WDS domain is underlined in area (B); and the putative nuclear targeting signal is underlined in area (C). **(B)** Phylogenetic relationship of the SiASR4 protein and its homologs in grass. SiASR4 is highlighted by a dark pentagram.

A full-length ASR gene (Seita.5G463800) was isolated from foxtail millet and designated *SiASR4*. Sequence analysis revealed that the full-length *SiASR4* cDNA has a 309-bp opening reading frame (ORF), encoding 102 amino acids, with a predicted molecular mass of 11.5 kDa and an isoelectric point of 10.57. BLASTX analysis showed that SiASR4 has two highly conserved regions: A small N-terminal consensus of 12 amino acids containing a stretch of four His residues, which is typical of zinc-binding regions, and a longer C-terminal region of 80 amino acids with an ABA/WDS domain and a putative signal for nuclear targeting (Figure 1A). Phylogenetic analysis indicated that the closest homolog to *SiASR4* in grass species is the green grass ASR protein, followed by rice and switch grass ASR clones. Here, we chose one of the closest homologs to *SiASR4* from each grass species as a representative. As shown in Figure 1B, the results suggested that the *SiASR4* sequence obtained in this study is a member of the ASR family of foxtail millet.

### Expression of the *SiASR4* gene in different tissues

**Figure 2:**
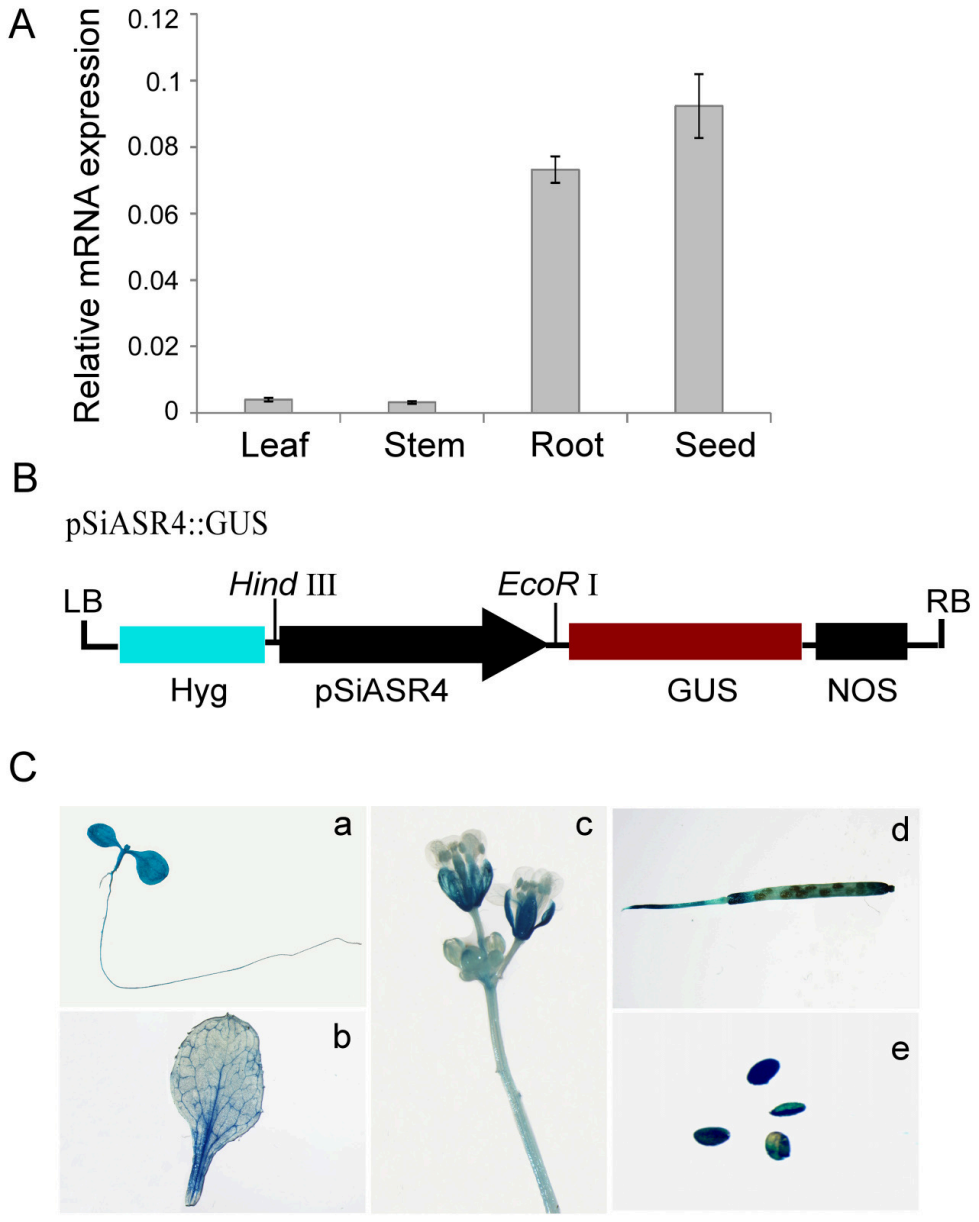
**Expression pattern of the *SiASR4* gene in different tissues. (A)** The transcription levels of *SiASR4* in different tissues of Jigu 11 foxtail millet seedlings. Foxtail millet actin (AF288226) served as a normalization control. **(B)** Diagram of the pSiASR4::GUS construct. **(C)** Spatial and temporal characterization of *SiASR4* through analysis of *SiASR4* promoter activity in pSiASR4::GUS transgenic *Arabidopsis*. (a) Five-day-old seedlings; (b) rosette leaf; (c) stem and flowers; (d) silique; (e) mature seeds.

We examined the expression levels of *SiASR4* in different foxtail millet tissues via qRT-PCR. SiASR4 was expressed in the leaves, stems, roots, and mature seeds, and the transcript levels were highest in mature seeds, followed by the roots, while the lowest level was detected in the leaves and stems (Figure 2A). We also isolated the putative *SiASR4* promoter region and confirmed it by sequencing. To characterize the temporal and spatial expression patterns of *SiASR4*, the GUS gene was driven by the *SiASR4* promoter and transformed into *Arabidopsis* (Figure 2B). GUS staining of transgenic *Arabidopsis* revealed GUS activity in the cotyledons, hypocotyls, stems, leaves, roots, flowers, and seeds, similar to foxtail millet, which indicated that *SiASR4* is a constitutively expressed gene (Figure 2C).

### *SiASR4* transcription is induced by PEG, ABA, and NaCl

**Figure 3:**
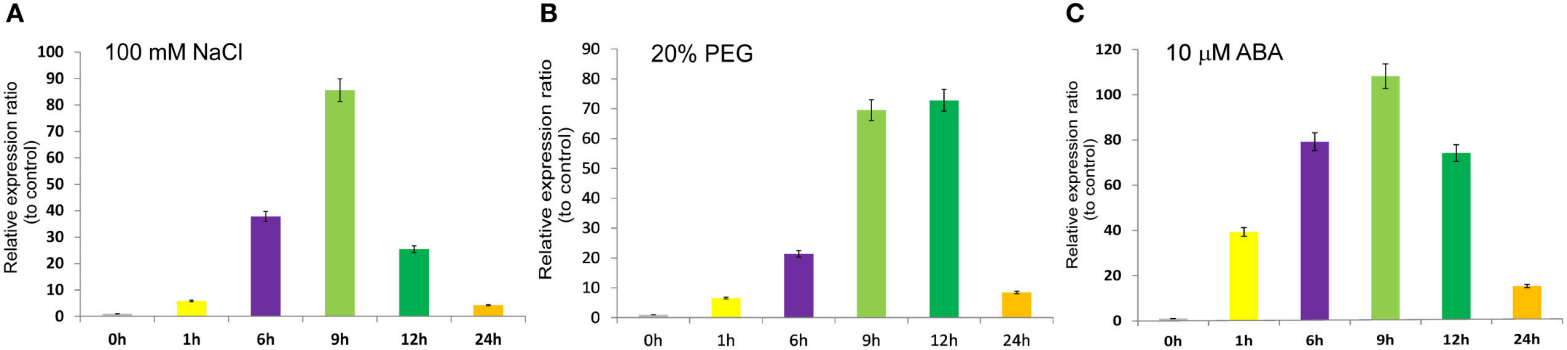
**Expression pattern of *SiASR4* in response to different stress treatments**. Two-week-old foxtail millet seedlings were treated independently with **(A)** 100 mM NaCl, **(B)** 20% (m/v) PEG6000 and **(C)** 10 μM ABA for the indicated times. Transcription levels were analyzed via qRT-PCR. Data are presented as the mean ± SEM (*n* = 3).

To determine the response of *SiASR4* to abiotic stress, PEG, NaCl, and ABA treatments were applied. Then, qRT-PCR was performed, and the results showed that the expression levels of *SiASR4* increased in response to 20% PEG6000, 100 mM NaCl and 10 μM ABA treatment (Figure 3). In response to NaCl and ABA treatment, *SiASR4* transcription increased after 1 h and peaked at 9 h (Figures 3A,C). In response to PEG treatment, *SiASR4* expression was induced to peak levels at 12 h and was maintained at similarly high levels at 9 h (Figure 3B). The level of *SiASR4* transcription decreased dramatically after 24 h of treatment. These results indicated that *SiASR4* may be involved in salt and drought stress responses via an ABA-dependent signaling pathway.

### Subcellular localization and transcriptional activation of *SiASR4*

**Figure 4:**
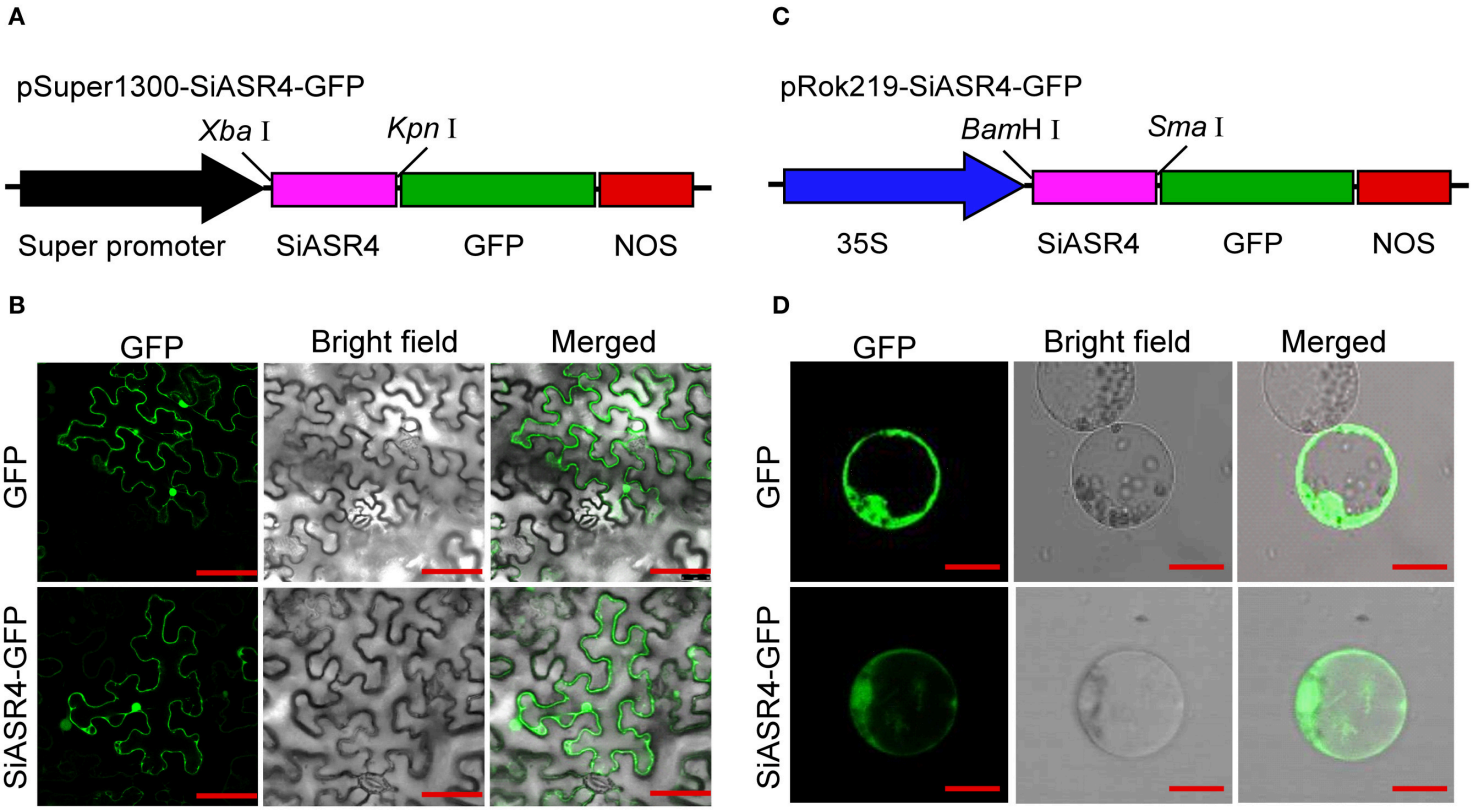
**Subcellular localization of *SiASR4*. (A)** Diagram of the pSuper1300-SiASR4-GFP construct. **(B)** Subcellular localization of GFP and the SiASR4-GFP fusion protein in *N. benthamiana* leaf cells. Bar = 100 μm. **(C)** Diagram of the pRok219-SiASR4-GFP construct. **(D)** Subcellular localization of GFP and SiASR4-GFP fusion protein in foxtail millet protoplasts. Bar = 20 μm.

To investigate the subcellular localization of SiASR4, a transient expression assay was performed with the SiASR4-GFP fusion protein and GFP alone (as a control) in *N. benthamiana* leaves. The fluorescence of SiASR4-GFP was distributed throughout whole cells in *N. benthamiana* leaves (Figures 4A,B), as was that of GFP. To confirm this result, the GFP and SiASR4-GFP fusion genes driven by the CaMV 35S promoter were transiently expressed in foxtail millet protoplasts. The results obtained in protoplasts showed that SiASR4 localized to the cell nucleus, cytoplasm and cytomembrane (Figures 4C,D).

To investigate the transcriptional activity of SiASR4, the yeast transcriptional activation system was used. Yeast cells were transformed with the pBD-GAL4-SiASR4 plasmid. All transformants grew well on SD/Trp-medium, but none of them grew on SD/Trp-/His-medium (Figure S1). These results indicated that SiASR4 lacked transcriptional activity in yeast cells.

### *SiASR4* improves abiotic stress tolerance in transgenic *Arabidopsis*

**Figure 5:**
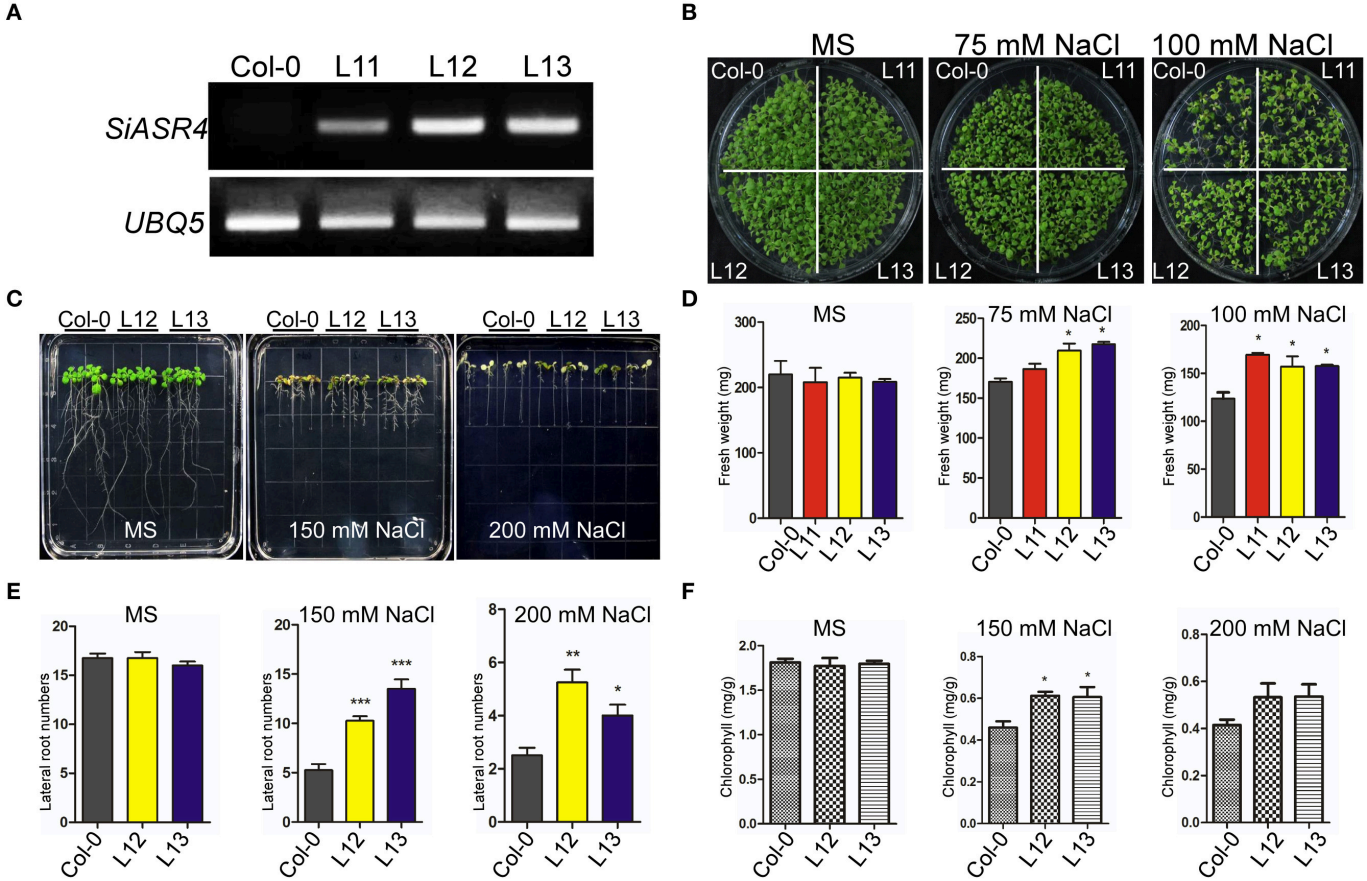
**Overexpression of *SiASR4* improves salt stress tolerance during the germination and young seedling stages of *Arabidopsis*. (A)** Expression levels of *SiASR4* in transgenic *Arabidopsis* and Col-0 *Arabidopsis* determined through semi-qRT-PCR analysis. **(B)** Analysis of the germination of Col-0 and transgenic *Arabidopsis*. Seedlings were germinated and grown for 10 days on MS medium containing 0, 75, or 100 mM NaCl. **(C)** The salt stress tolerance of Col-0 and transgenic *Arabidopsis* was assessed in the young seedling stage. Five-day-old seedlings grown on MS medium were transferred to MS medium containing different concentrations of NaCl for 7 days. **(D)** Fresh weight of the seedlings shown in **(B)**. All samples were measured in triplicate. **(E)** Fresh weight of the seedlings shown in **(C)**. All samples were measured in triplicate. **(F)** Analysis of the chlorophyll content of the seedlings in **(C)**. Each data point comprises three replicates. For **(D–F)**, error bars represent ±*SD*. ^*^, ^**^, and ^***^ indicate statistical significance at *P* < 0.05, 0.01, and 0.001 (Student's *t*-test), respectively.

To assess the function of *SiASR4* in stress tolerance, transgenic *Arabidopsis* plants overexpressing *SiASR4* under the control of the CaMV 35S promoter were generated. *SiASR4* transgenic lines were obtained using a floral dip method. Transgenic T1 seedlings were selected on MS medium supplemented with 50 μg/ml kanamycin and then confirmed by PCR. Finally, 18 *SiASR4*-overexpressing transgenic *Arabidopsis* plants were obtained and three independent homozygous *SiASR4* T3 lines (L11, L12, and L13) with high expression levels were chosen for further functional analyses (Figure 5A).

Under normal conditions, there were no obvious differences in fresh weight between transgenic and wild-type (WT) *Arabidopsis* in either the seed germination or growth stage. However, the transgenic plants grew better than wild-type plants on MS medium supplemented with 75 or 100 mM NaCl (Figure 5B). They displayed larger cotyledons than WT plants, and the fresh weights of the transgenic lines were significantly greater than those of WT plants (Figures 5B,D). However, when grown on MS medium containing different concentrations of mannitol or ABA, the WT and transgenic lines did not exhibit an obvious difference (Figures S2A,B). These results indicated that *SiASR4* can enhance salt stress tolerance in transgenic *Arabidopsis* in both the seed germination and growth stages.

To evaluate the effect of abiotic stress during the seedling stage, 5-day-old *Arabidopsis* grown in normal medium were transferred to MS medium containing different concentrations of NaCl, mannitol and ABA for 7 days. There were no obvious differences between the transgenic and WT plants under normal conditions or in response to mannitol and ABA (Figures S3A,B). When grown on the MS medium containing 150 or 200 mM NaCl, the transgenic plants showed more robust growth than WT plants (Figure 5C), which was characterized by a significant increase in the number of lateral roots and chlorophyll content (Figures 5E,F). These results showed that WT plants were more sensitive than transgenic plants to salt stress during the seedling stage.

**Figure 6:**
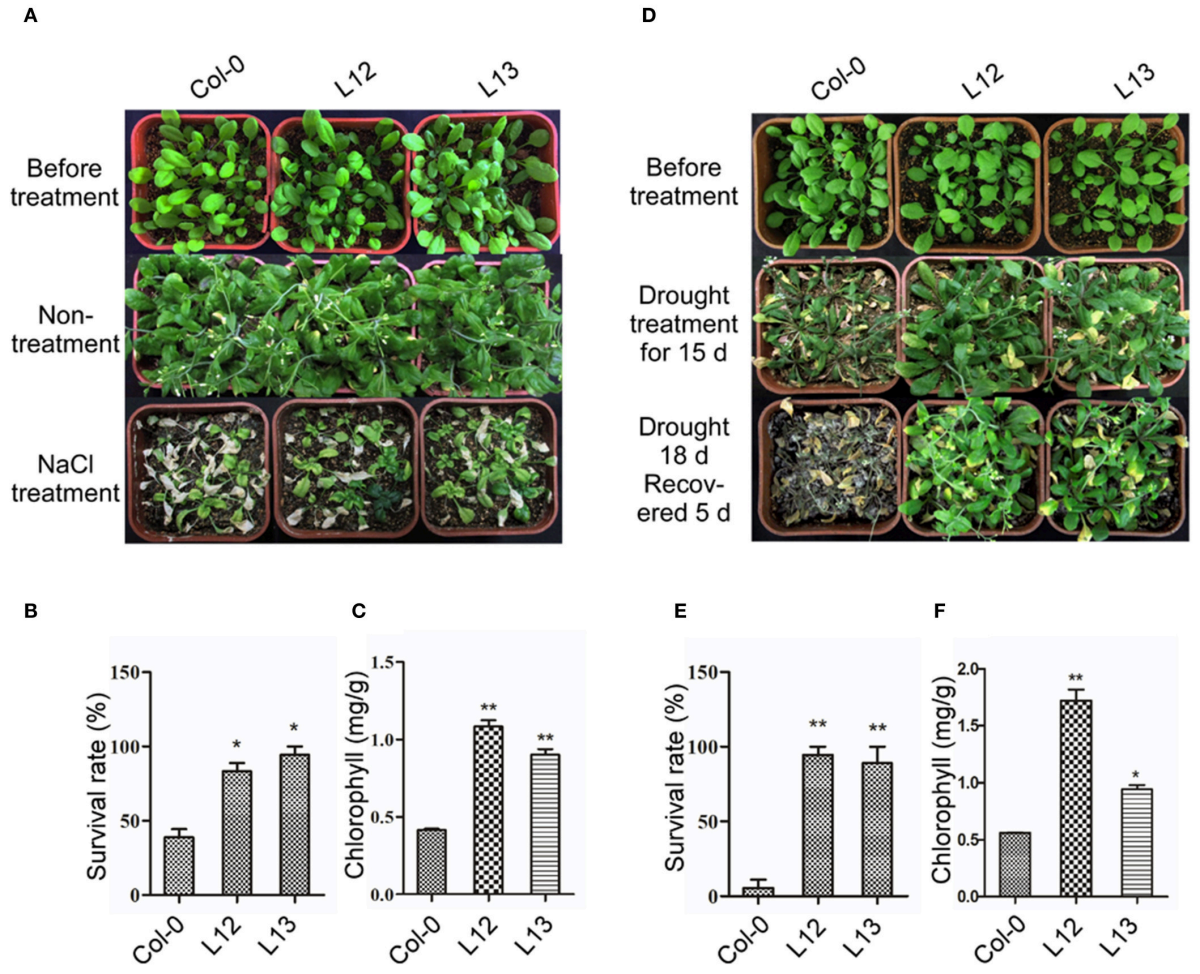
**Overexpression of *SiASR4* improves salt and drought stress tolerance in *Arabidopsis* in soil. (A)** NaCl stress tolerance of Col-0 and transgenic *Arabidopsis*. Three-week-old *Arabidopsis* were treated with 400 mM NaCl for 14 days. **(B)** Analysis of the survival rate of Col-0 and transgenic *Arabidopsis* shown in **(A)**. **(C)** Analysis of the chlorophyll content of the seedlings shown in **(A)**. **(D)** Drought tolerance of Col-0 and transgenic *Arabidopsis*. Three-week-old *Arabidopsis* were not watered for 18 days and were then rewatered for 5 days. **(E)** Analysis of the survival rate of Col-0 and transgenic *Arabidopsis* shown in **(D)**. **(F)** Analysis of the chlorophyll content of the seedlings shown in **(D)**. The experiments comprised three replicates. For **(B,C,E,F)**, error bars represent ±*SD*. ^*^ and ^**^ indicate statistical significance at *P* < 0.05 and 0.01 (Student's *t*-test), respectively.

To further assess the function of *SiASR4* during salt and drought tolerance, the corresponding treatments were applied to 3-week-old transgenic and WT plants grown under normal conditions. After irrigation with water supplemented with 400 mM NaCl for 14 days, more WT than transgenic plants were bleached (Figure 6A). Approximately 38% of the WT plants survived, while the survival rates of the transgenic lines were greater than 80% (Figure 6B). The chlorophyll content was increased in the transgenic lines compared with WT plants during salt stress treatment (Figure 6C). For the drought stress treatment, the plants were not irrigated for 18 days. When the plants were rewatered and grown for 5 days, almost all of the WT seedlings died (Figure 6D), while approximately 90% of transgenic plants survived (Figure 6E). The chlorophyll content was decreased by at least 50% in WT plants compared with the transgenic lines (Figure 6F). These results indicated that *SiASR4* could enhance drought and salt tolerance in soil conditions. Considering the obtained results together, we concluded that *SiASR4* functions in the stress response of *Arabidopsis*.

### Overexpression of *SiASR4* increases the abiotic stress tolerance of foxtail millet

**Figure 7:**
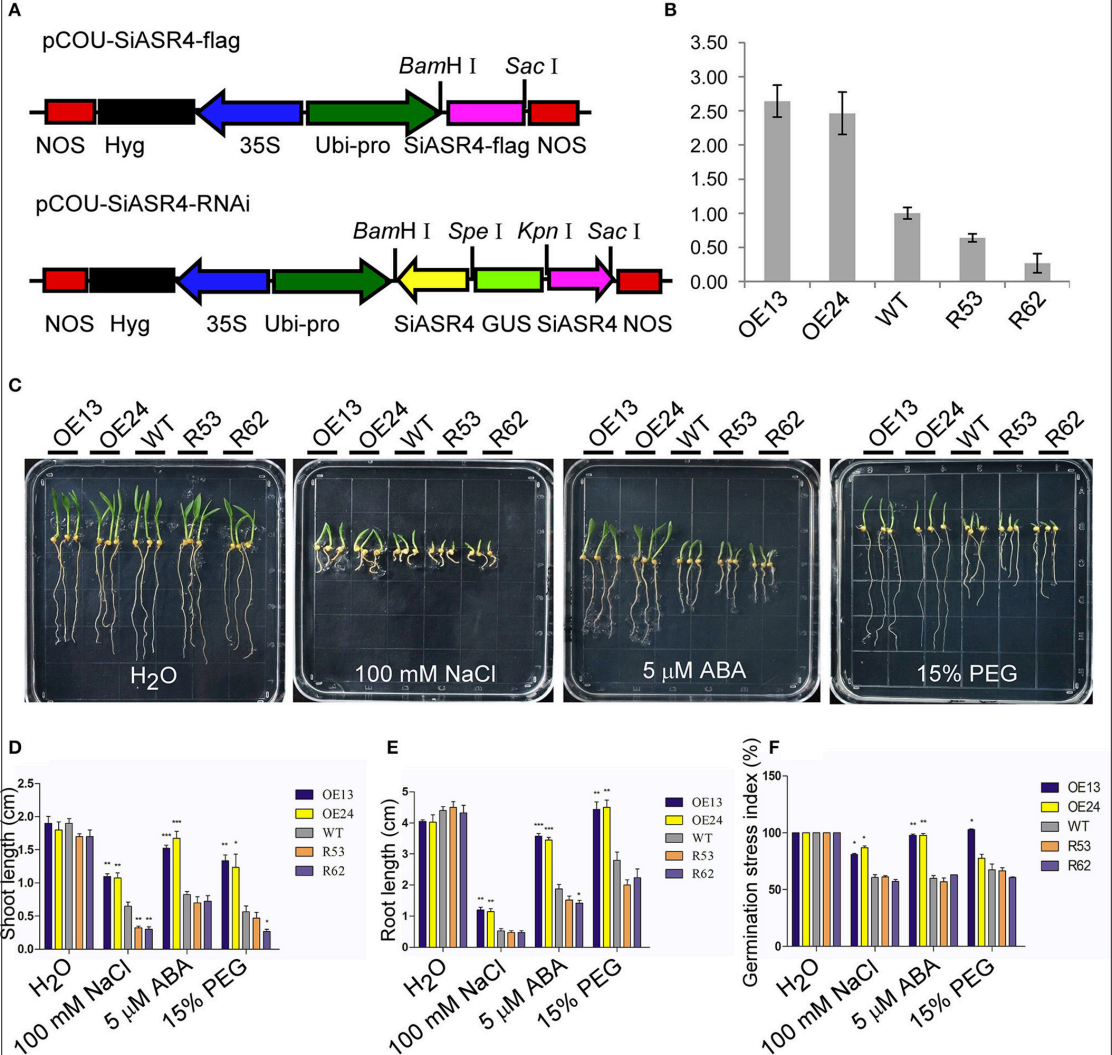
**Assessment of NaCl, PEG and ABA stress on *SiASR4*-overexpressing and *SiASR4*-RNAi foxtail millet during the germination stage. (A)** Diagram of the T-DNA region of the binary vectors pCOU-SiASR4-flag and pCOU-SiASR4-RNAi. **(B)** Expression levels of WT and transgenic foxtail millet determined via qRT-PCR. Foxtail millet actin (AF288226) served as a normalization control. **(C)** Treatment with various types of abiotic stresses and ABA in transgenic and WT foxtail millet during the germination stage. The seeds were germinated on filter paper containing H_2_O, 100 mM NaCl, 5 μM ABA, or 15% PEG. The experiment comprised three replicates. **(D,E)** Analysis of shoot and root length in **(C)** on day six. **(F)** Analysis of the germination stress index of WT and transgenic foxtail millet. For **(D–F)**, error bars represent ±*SD*. ^*^, ^**^, and ^***^ indicate statistical significance at *P* < 0.05, 0.01, and 0.001 (Student's *t*-test), respectively.

To further investigate the role of *SiASR4*, we obtained *SiASR4*-overexpressing and RNAi transgenic foxtail millet lines confirmed by PCR. Based on the qRT-PCR results, two overexpressing lines (OE13 and OE24) and two RNAi transgenic lines (RNAi53 and RNAi62) were chosen for further analysis (Figures 7A,B).

First, we examined abiotic stress tolerance in the transgenic lines during the germination stage. Transgenic and WT foxtail millet seeds were germinated under different treatments. There were no apparent differences in shoot or root length between the transgenic and WT lines exposed to water for 6 days. When germinated in 5 μM ABA, 100 mM NaCl, or 15% PEG for 6 days, the growth of the *SiASR4*-overexpressing lines was significantly better than that of WT plants, and the shoot and root lengths of *SiASR4*-overexpressing lines were dramatically longer compared with WT. However, there was no obvious suppression of the *SiASR4*-RNAi lines compared with WT, except with respect to shoot length after treatment with 100 mM NaCl (Figures 7C–E). We further analyzed the germination stress index after 8 days. The germination stress index was higher in the *SiASR4*-overexpressing lines compared with WT, but no differences were observed between *SiASR4*-RNAi and WT (Figure 7F). These results implied that overexpression of *SiASR4* in foxtail millet enhanced resistance to drought and salt, and these plants were insensitive to ABA during the germination stage.

**Figure 8:**
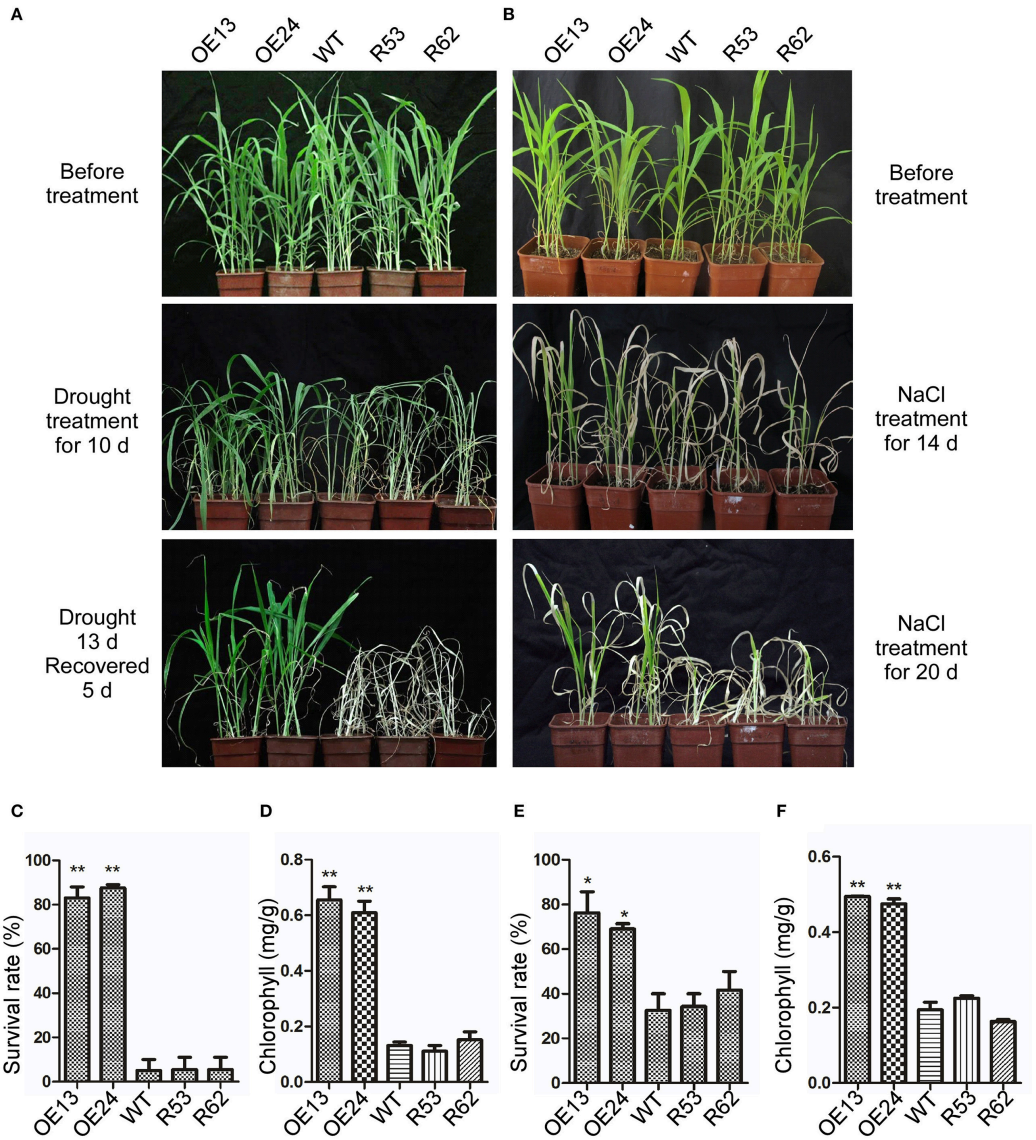
**Overexpression of *SiASR4* improves drought and salt stress tolerance in foxtail millet in soil. (A)** Drought tolerance of WT, *SiASR4*-overexpressing and *SiASR4*-RNAi foxtail millet. Three-week-old plants were not watered for 13 days, followed by watering for 5 days. **(B)** Salt tolerance of WT, *SiASR4*-overexpressing and *SiASR4*-RNAi foxtail millet. Three-week-old plants were treated with 400 mM NaCl for 20 days. **(C,E)** Survival rates of WT, *SiASR4*-overexpressing and *SiASR4*-RNAi foxtail millet shown in **(A)** and **(B)**. **(D,F)** Analysis of the chlorophyll content of seedlings in **(A)** and **(B)**. For **(C–F)**, error bars represent ±*SD*. ^*^ and ^**^ indicate statistical significance at *P* < 0.05 and 0.01 (Student's *t*-test), respectively.

To analyze the performance of transgenic lines in response to drought and salt treatment in soil, 3-week-old *SiASR4*-overexpressing, *SiASR4*-RNAi and WT seedlings were subjected to the corresponding treatments. For drought stress treatment, seedlings were grown under water stress conditions for 13 days and then rewatered and grown under normal conditions for 5 days (Figure 8A). More than 80% of the overexpressing lines survived, while the survival rate of the WT lines was ~10% (Figure 8C). For salt stress treatment, seedlings were irrigated with water supplemented with 400 mM NaCl for 20 days (Figure 8B). Approximately 32% of the WT plants survived, while the survival rates of the overexpressing lines were greater than 68% (Figure 8E). In addition, the overexpressing lines displayed higher chlorophyll content than WT plants. However, there were no obvious differences between the RNAi and WT lines (Figures 8D,F).

### *SiASR4* regulates ROS scavenger-associated and stress-responsive gene expression

**Figure 9:**
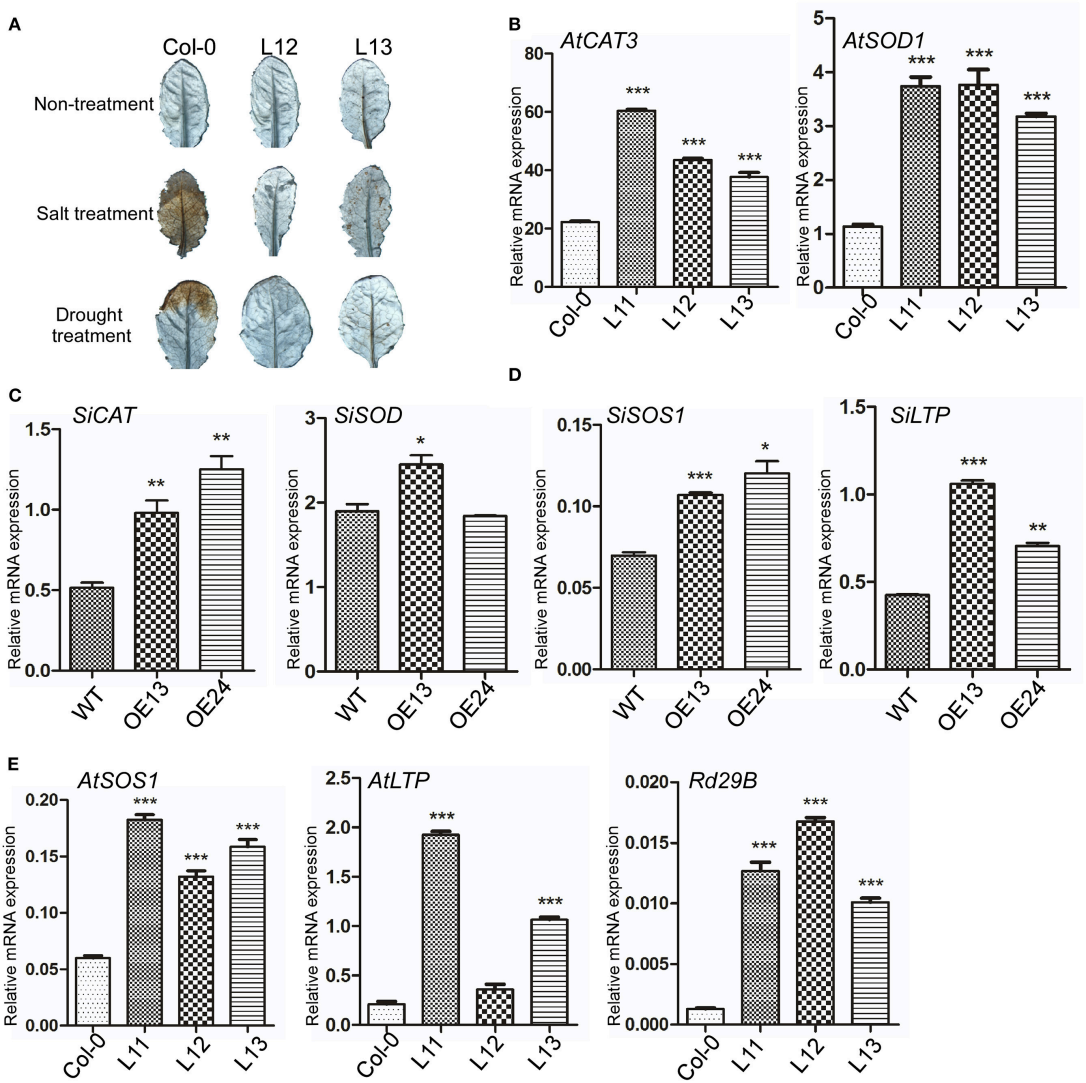
***SiASR4* regulates stress-responsive and ROS scavenger-associated gene expression levels in transgenic *Arabidopsis* and foxtail millet. (A)** Overexpression of *SiASR4* reduces ROS accumulation in *Arabidopsis* in response to drought and salt stress treatment. Three-week-old *Arabidopsis* were not watered and were treated with 400 mM NaCl for 6 days, respectively. The fourth and fifth rosette leaves were harvested and then stained with DAB to detect H_2_O_2_ production. **(B)** Expression of ROS scavenger-associated genes in *Arabidopsis*. **(C)** Expression of ROS scavenger-associated genes in foxtail millet. **(D)** Expression of stress-responsive genes in foxtail millet. **(E)** Expression of stress-responsive genes in *Arabidopsis*. *UBQ5* (At3g62250) was used as a normalization control in Arabidopsis. *Actin* (AF288226) was used as a normalization control in foxtail millet. Error bars represent ±SD. ^*^, ^**^, and ^***^ indicate statistical significance at *P* < 0.05, 0.01, and 0.001 (Student's *t*-test), respectively.

Abiotic stresses, such as drought and high-salinity, can induce the production of ROS (Foreman et al., 2003). It has been reported that *TaASR1* and *SbASR1* induce the transcription of ROS scavenger-associated genes and reduce the accumulation of ROS (Hu et al., 2013; Tiwari et al., 2015). To analyze the role of *SiASR4* in ROS scavenging in transgenic *Arabidopsis*, 3-week-old Col-0 and transgenic *Arabidopsis* grown in pots were not watered and were treated with salt for 6 days, respectively. The fourth and fifth rosette leaves were then harvested and stained with DAB to detect H_2_O_2_ production. The results showed that the levels of DAB staining in H_2_O_2_ in rosette leaves were similar among the Col-0 and transgenic lines under normal conditions. After drought and salt treatment, *SiASR4* transgenic *Arabidopsis* accumulated less H_2_O_2_ than Col-0 *Arabidopsis* (Figure 9A), indicating that overexpression of *SiASR4* reduces ROS accumulation in transgenic *Arabidopsis*.

To analyze the expression of ROS scavenger-associated genes in transgenic *Arabidopsis*, the *AtCAT3 and AtSOD1* genes, which are involved in ROS detoxification, were selected. Without stress treatment, the expression of *AtCAT3* and *AtSOD1* was increased significantly in transgenic compared with WT *Arabidopsis* (Figure 9B). We further analyzed the expression levels of *SiCAT* and *SiSOD* in *SiASR4*-overexpressing foxtail millet. The results showed that the ROS scavenger-associated genes were upregulated in *SiASR4*-overexpressing foxtail millet (Figure 9C). Furthermore, we analyzed expression of stress-responsive genes, the *SiSOS1* and *SiLTP* genes of foxtail millet, and the *AtSOS1, AtLTP3*, and *Rd29B* genes of *Arabidopsis* by qRT-PCR. The results showed that the expression of these genes was increased markedly in *SiASR4*-overexpressing foxtail millet and *Arabidopsis* under normal conditions (Figures 9D,E). These results indicated that overexpression of *SiASR4* in foxtail millet and *Arabidopsis* enhances the expression of ROS scavenger-associated and stress-responsive genes.

### SiARDP binds to DRE motifs in the promoter of *SiASR4*

**Figure 10:**
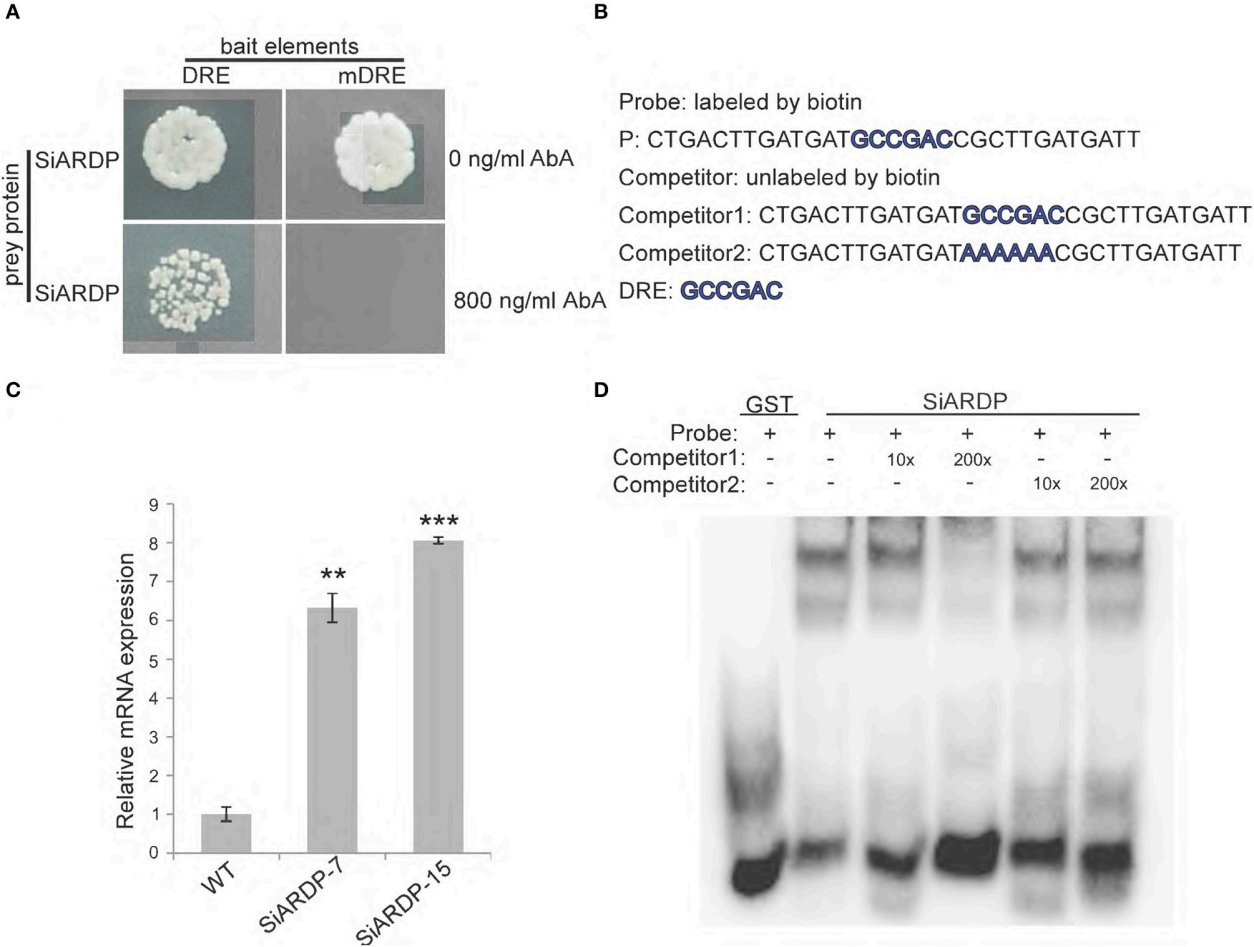
**SiARDP binds to DRE element in the *SiASR4* promoter and regulates *SiASR4* expression. (A)** Yeast one-hybrid assays showing that SiARDP binds to the *SiASR4* promoter. **(B)** The probe and competitor probe sequences used for EMSA. **(C)** SiARDP binding to the DRE element is shown in the presence of different concentrations of competitors. **(D)** Expression levels of *SiASR4* in *SiARDP* transgenic foxtail millet. Error bars represent ±*SD*. ^**^ and ^***^ indicate statistical significance at *P* < 0.01 and 0.001 (Student's *t*-test), respectively.

Because *SiASR4* responded to drought and salt stress and ABA treatment, we analyzed the *SiASR4* promoter and identified five DRE motifs. *SiARDP*, which we had previously cloned from foxtail millet, is a transcription factor that can bind to DRE elements (Li et al., 2014). To assess whether *SiARDP* is involved in the regulation of *SiASR4 in vivo* and *in vitro*, yeast one-hybrid assays and EMSAs were performed. One bait sequence in the *SiASR4* promoter containing the DRE element (GCCGAC) was inserted into a bait plasmid. Simultaneously, a mutated DRE element (AAAAAA) sequence was also prepared (Table S1) and transferred plasmids to yeast cells; 800 ng/mL AbA suppressed the basal expression. Additionally, *SiARDP* was inserted into the pGADT7-AD plasmid, and the resulting plasmid was then transformed into the bait strain or mutated strain. The bait strain yeast cells transformed with pGADT7-SiARDP could grow on medium lacking Leu and containing 800 ng/mL AbA, but the mutant strain yeast cells with pGADT7-SiARDP did not grow (Figure 10A).

We also conducted an EMSA experiment. The sequence containing the DRE element was used as a probe (P). Full-length SiARDP was expressed as a glutathione S-transferase (GST) fusion protein in *E. coli*. The results showed that SiARDP could bind to the probe. The addition of different concentrations of unlabeled probes reduced the binding signal to various degrees, but the addition of mutant probes did not reduce the binding signal (Figures 10B,D). These results suggested that SiARDP specifically bound to the DRE core element in the *SiASR4* promoter.

To further analyze whether *SiASR4* is regulated by *SiARDP in vivo*, we examined the expression level of *SiASR4* in *SiARDP*-overexpressing foxtail millet. Two independent homozygous *SiARDP*-overexpressing T3 lines were chosen for the analysis. qRT-PCR showed that the expression of *SiASR4* was upregulated at least 6-fold in *SiARDP* transgenic foxtail millet (Figure 10C). Based on these results, *SiASR4* was likely regulated by *SiARDP* in foxtail millet.

## Discussion

Since the first discovery of tomato ASR1, more than 20 years have passed. An increasing number of ASR families have been characterized in various plants. The functions of these proteins are involved in the responses to ABA and abiotic stress as well as the process of fruit ripening (Kalifa et al., 2004). However, foxtail millet, which is an important crop in China, has been less well-studied than other crops, and the ASR regulatory pathway in this species has been unclear. In the present study, the ASR gene family in foxtail millet was analyzed using the Phytozome BLAST algorithm and five *SiASR* genes were identified (Figure 1B). However, Feng et al. (2016) reported that there are six *SiASR* genes in foxtail millet. This difference may be due to the different version of Phytozome. The newest version of Phytozome v11 was used in the present work. Further analysis showed that these five *SiASR* genes expressions were induced by ABA treatment as well as NaCl, PEG and low temperature (4°C) treatments. What's more, *SiASR4* transcription was highly induced in response to abiotic stresses (data not shown). As a result, the *SiASR4* gene was cloned and the role of *SiASR4* during abiotic stress and the related regulatory pathway were analyzed.

Most ASR proteins exhibit similar structural features, harboring a zinc-binding domain at the N-terminus and a putative nuclear targeting signal at the C-terminus (Cakir et al., 2003). Different ASR proteins show different subcellular localizations. We found that the SiASR4 protein also contains a putative nuclear targeting signal and is distributed throughout whole cells (Figure 4). This result suggested that SiASR4 may play a dual role as a transcription factor and a protective protein. In *SiASR4*-overexpressing plants, the expression levels of stress-responsive and ROS scavenger-associated genes were upregulated under normal conditions. This finding suggested that *SiASR4* may function as a transcription factor. Unfortunately, transcriptional activation of SiASR4 was not detected in yeast (Figure S1). In plants, the transcription of some transcription factors must be regulated by phosphorylation. Phosphorylation site prediction for SiASR4 showed that the SiASR4 protein exhibited three phosphorylation sites (data not shown). We speculate that in yeast cells, SiASR4 may not be activated by phosphorylation. However, whether *SiASR4* acts as a transcription factor remains to be investigated.

ASR proteins play important roles in abiotic stress tolerance in most plants, as observed for *OsASR1, ZmASR1*, and *TaASR1* (Virlouvet et al., 2011; Hu et al., 2013; Arenhart et al., 2016). *SiASR4*, which is reported herein, was also found to enhance abiotic stress resistance in transgenic plants. Overexpression of *SiASR4* improved drought and high-salinity stress resistance in transgenic *Arabidopsis*. Furthermore, *SiASR4* enhanced drought and salt tolerance capabilities during *Arabidopsis* development (Figures 5, 6). ASR proteins have been shown to participate in the ripening process (Cakir et al., 2003). Our results are consistent with a previous report, and the gene was found to show greater functionality in the late growth stage. The *Arabidopsis* genome lacks ASR homolog (Wong et al., 2006), whereas *SiASR4* functions in *Arabidopsis*. This result suggested the presence of similar pathways in transgenic *Arabidopsis*. Furthermore, overexpression of *SiASR4* enhanced drought and salt tolerance in foxtail millet (Figures 7, 8), which is consistent with the expression patterns of *SiASR4* observed in response to NaCl and PEG treatment (Figure 3). However, the *SiASR4*-RNAi lines were not markedly more sensitive to drought and salt stress than WT plants. This finding may be explained by functional redundancy of the *SiASR* gene family members.

ABA mediates the adaptation of plants to abiotic stress, such as drought and high salinity (Busk and Pagès, 1998), and many ASR genes are induced by ABA treatment (Ricardi et al., 2014). In the present study, *SiASR4* transgenic *Arabidopsis* did not respond to exogenous ABA treatment in either the germination or seedling stage (Figures S2B, S3B), whereas *SiASR4*-overexpressing foxtail millet was more insensitive to exogenous ABA than WT plants (Figure 7). These findings indicated that *SiASR4* participates in an ABA-dependent pathway during the abiotic stress response in foxtail millet. In *SiASR4* transgenic *Arabidopsis, SiASR4* did not respond to ABA treatment and may therefore belong to other signaling pathways that play a role in abiotic stress.

In previous studies, *LTP* and *Rd29B* were found to be induced by drought and salt stress (Torres-Schumann et al., 1992; Trevino and O'Connell, 1998; Fu et al., 2014) and *SOS1* to be induced by salt stress (Shi et al., 2000). Overexpression of *SiASR4* induced the expression of stress-responsive genes in transgenic *Arabidopsis* and foxtail millet (Figures 9D,E). There was a positive relationship between stress-responsive genes and stress tolerance in *SiASR4*-overexpressing plants. These results indicated that *SiASR4* enhanced stress tolerance by regulating these genes. Salinity and drought induce the generation of ROS (Singh et al., 2016). The wheat ASR gene *TaASR1* mediates resistance to ROS-mediated injury during drought stress (Hu et al., 2013). Overexpression of *SiASR4* reduced the accumulation of H_2_O_2_ detected by DAB staining during drought and salt treatment. Overexpression of *SiASR4* also induced the expression of ROS scavenger-associated genes in transgenic *Arabidopsis* and foxtail millet (Figures 9A–C). These data indicated that drought and salt stress tolerance in *SiASR4*-overexpressing lines may be correlated with the ability to reduce ROS accumulation. However, further investigation is needed to validate this hypothesis.

To gain further insight into the molecular mechanism underlying the function of *SiASR4*, the *cis*-element of the *SiASR4* promoter was identified. The promoter region of *SiASR4* was found to contain five DRE core elements and to be induced by different stress treatments. DREB transcription factors bind to DRE elements and are important in abiotic stress signal transduction pathways. *SiARDP* has been reported to be involved in ABA-dependent signaling pathway underlying salt and drought stress (Li et al., 2014). In the present study, *SiASR4* transcription was shown to be upregulated by drought, salt, and exogenous ABA treatments, consistent with the expression pattern of *SiARDP* observed in response to abiotic stress treatment. Yeast one-hybrid assays and EMSAs showed that *SiARDP* binds to the DRE element (GCCGAC) of the *SiASR4* promoter. *SiASR4* transcription was induced in *SiARDP*-overexpressing foxtail millet (Figure 10). These results indicated that *SiASR4* may be regulated by *SiARDP* in foxtail millet during drought and salt stress via an ABA-dependent pathway.

In summary, the results of this study demonstrated that overexpression of *SiASR4* enhanced salt and drought stress in *Arabidopsis* and foxtail millet. These processes may activate the antioxidant system and regulate the expression of stress-related genes. Furthermore, *SiARDP* can bind to the DRE core element of the *SiASR4* promoter and activate the expression of *SiASR4*. Although the precise mechanism by which *SiASR4* enhances abiotic stress must be confirmed in further investigations, our results demonstrated that *SiASR4* plays an important role in plant salt and drought stress resistance.

## Author contributions

JY, JL, and YD conceived and designed the experiments. JL, YD, CL, and YP performed the experiments. JL and JY wrote the original manuscript. JY thoroughly revised the manuscript and finalized the manuscript. All authors read and approved the final manuscript.

## Funding

This work was supported by the National Basic Research Program of China (Grant No. 2012CB215301) and the National Transgenic Major Program of China (Grant No. 2014ZX08003-002).

### Conflict of interest statement

The authors declare that the research was conducted in the absence of any commercial or financial relationships that could be construed as a potential conflict of interest. The reviewer PS and handling Editor declared their shared affiliation, and the handling Editor states that the process nevertheless met the standards of a fair and objective review.

## Supplementary material

Figure S1 **Transcriptional activation activity assay for SiASR4**. + indicates transformants carrying the pGAL4 vector, as the positive control. – indicates transformants carrying the pBD-GAL4 vector, as the negative control.

Figure S2 **Assay for mannitol and ABA treatments in *SiASR4* transgenic *Arabidopsis* during the germination stage. (A)** Analysis of the germination of Col-0 and transgenic *Arabidopsis* during mannitol treatment. Seedlings were germinated and grown for 10 days on MS medium containing 0, 100, or 200 mM mannitol. **(B)** Col-0 and transgenic *Arabidopsis* were germinated under ABA treatment. The seedlings were germinated and grown for 10 days on MS medium containing 0, 0.5, or 1 μM ABA.

Figure S3 **Assay for mannitol and ABA stress in *SiASR4* transgenic *Arabidopsis* during the seedling stage. (A)** Analysis of mannitol tolerance in Col-0 and transgenic *Arabidopsis* during the young seedling stage. Five-day-old seedlings grown on MS medium were transferred to MS medium containing different concentrations of mannitol for 7 days. **(B)** Analysis of the ABA sensitivity of Col-0 and transgenic *Arabidopsis* during the young seedling stage. Five-day-old seedlings grown on MS medium were transferred to MS medium containing different concentrations of ABA for 7 days.

Table S1 **Primers and bait sequences used in this study**.
