# Supplementary figures and images for: SiASR4, the Target Gene of SiARDP from Setaria italica, Improves Abiotic Stress Adaption in Plants
Source: Front Plant Sci. 2017 Jan 12;7:2053. doi: 10.3389/fpls.2016.02053 (PMC5227095; doi:10.3389/fpls.2016.02053)

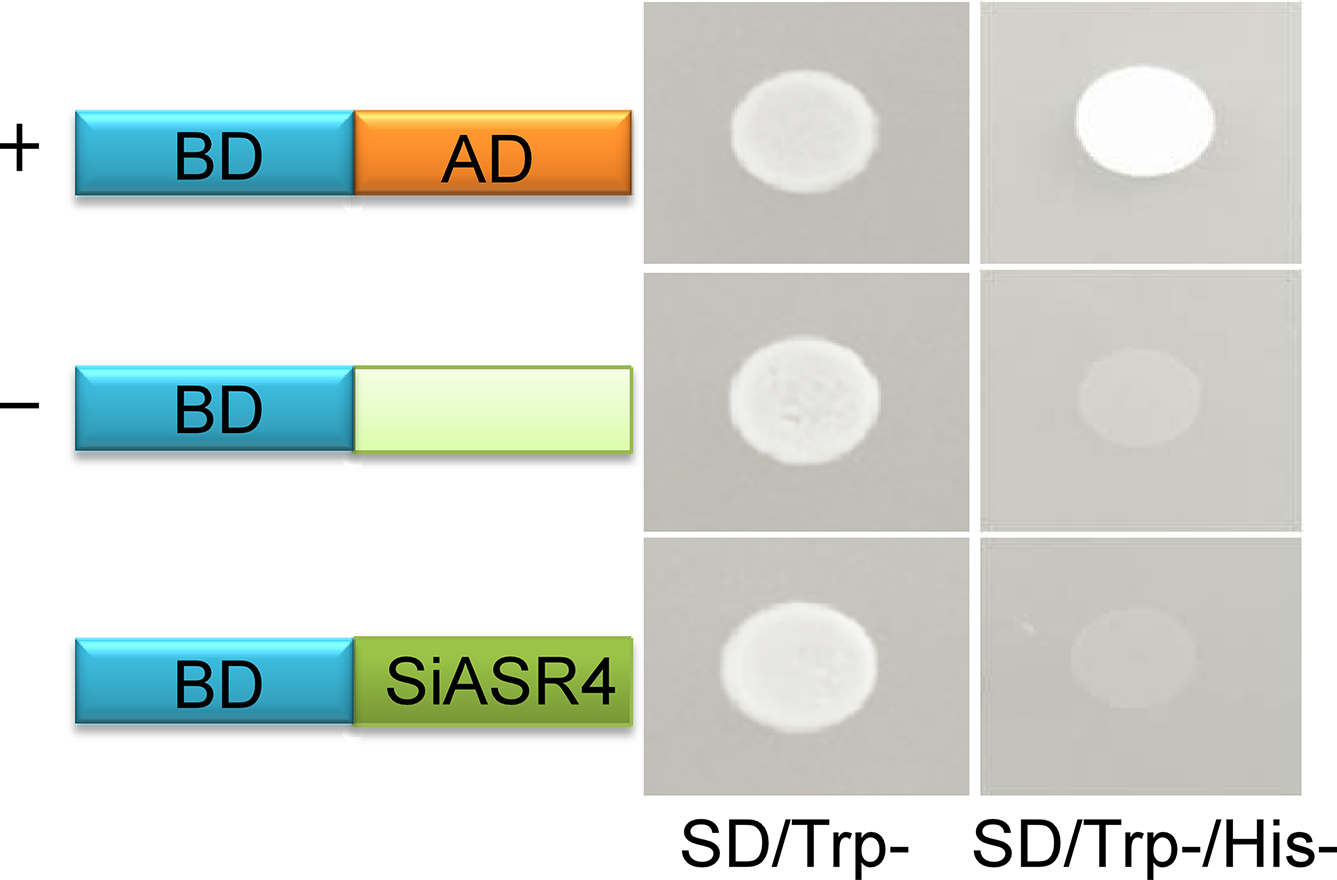

Supplement: Figure S1 — Transcriptional activation activity assay for SiASR4. + indicates transformants carrying the pGAL4 vector, as the positive control. – indicates transformants carrying the pBD-GAL4 vector, as the negative control. [file Image1.TIF]

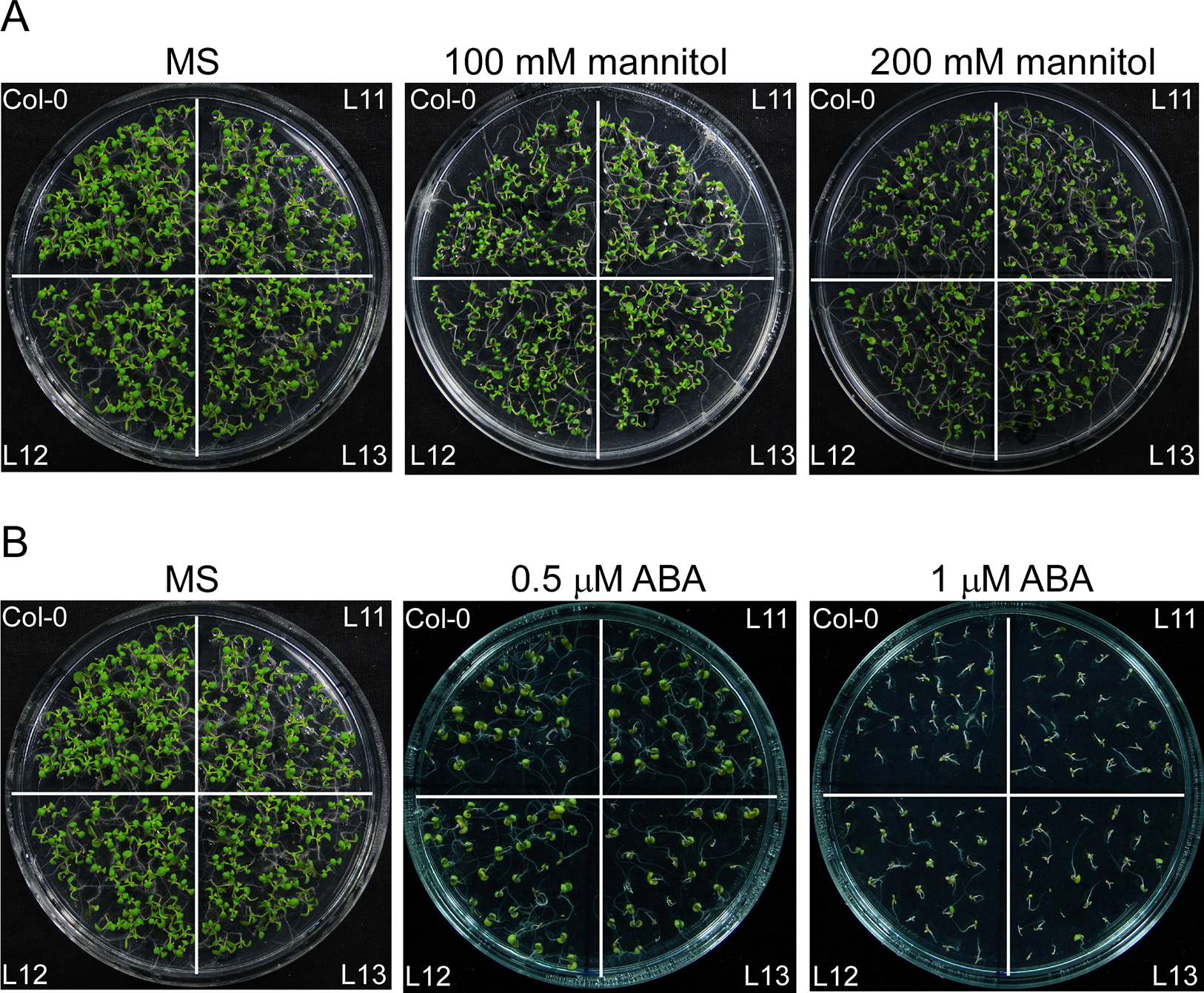

Supplement: Figure S2 — Assay for mannitol and ABA treatments in SiASR4 transgenic Arabidopsis during the germination stage. (A) Analysis of the germination of Col-0 and transgenic Arabidopsis during mannitol treatment. Seedlings were germinated and grown for 10 days on MS medium containing 0, 100, or 200 mM mannitol. (B) Col-0 and transgenic Arabidopsis were germinated under ABA treatment. The seedlings were germinated and grown for 10 days on MS medium containing 0, 0.5, or 1 μM ABA. [file Image2.TIF]

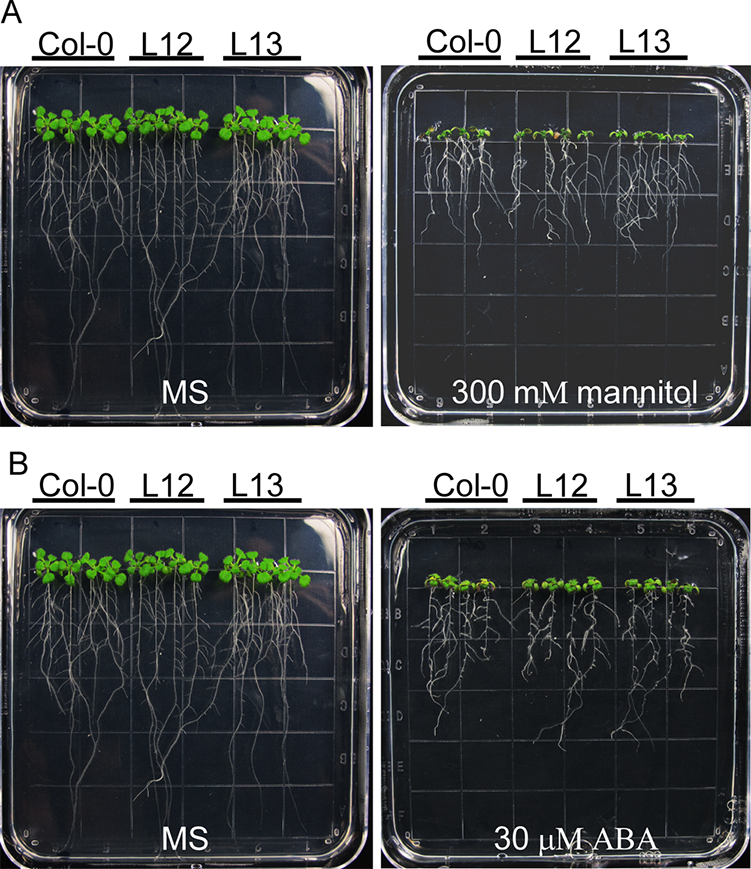

Supplement: Figure S3 — Assay for mannitol and ABA stress in SiASR4 transgenic Arabidopsis during the seedling stage. (A) Analysis of mannitol tolerance in Col-0 and transgenic Arabidopsis during the young seedling stage. Five-day-old seedlings grown on MS medium were transferred to MS medium containing different concentrations of mannitol for 7 days. (B) Analysis of the ABA sensitivity of Col-0 and transgenic Arabidopsis during the young seedling stage. Five-day-old seedlings grown on MS medium were transferred to MS medium containing different concentrations of ABA for 7 days. [file Image3.tif]
