# Supplementary material for: SiASR4, the Target Gene of SiARDP from Setaria italica, Improves Abiotic Stress Adaption in Plants
Source: Front Plant Sci. 2017 Jan 12;7:2053. doi: 10.3389/fpls.2016.02053 (PMC5227095; doi:10.3389/fpls.2016.02053)
Supplement: Table S1 — Primers and bait sequences used in this study. [file Table1.pdf]

Table S1 Primers and bait sequences used in this study		
Gene name	Forward primer	Reverse primer
SiASR4	5'-ATGGGTCACCACCACCACGA-3'	5'-ATGGTGGTGCTGGTGCC-3'
proSiASR4	5'-GTCGAGATATATGGATGGGTGA-3'	5'-CGATGAATTGTGTCTTGTGTTG-3'
SiASR4-OE	5'-TTTTGTTCGCTTGGTTGTGA-3'	5'-TCCGCCTTGTTCTTGTTCTT-3'
SiASR4-RNAi	5'-GCGGTCTAGCCTGGTCACTG-3'	5'-TTTATCCGTTCGTCCATTTGTA-3'
SiASR4 (qRT)	5'-ACCACCACCACGACAAGAACAAG-3'	5'-CTGCGACGGCACCGACCT-3'
AtCAT3	5'-GCGTTGAAACCTAACCCGAAAA-3'	5'-AAACCCTCCATGTGCCTGTAATCTT-3'
AtSOD1	5'-TGAGGGTGTTACGGGGACTATCT-3'	5'-GTGAAGGTGGCAGTTCCATCATCTC-3'
AtSOS1	5'-TCATCATCCTCACAATGGCTCTAA-3'	5'-ACCAACTTGCGTGGGACAACTTTA-3'
AtLTP3	5'-GGCTTTCGCTTTGAGGTTCTTC-3'	5'-ATGCTAACACCGCACTTTCCAG-3'
Rd29B	5'-GAGTGAAGGAGACGCAACAAGGG-3'	5'-GGTTTACCACCGAGCCAAGAAGT-3'
SiCAT	5'-TCAAGCCAAACCCAAAGACCAAT-3'	5'-CCCAGCGGCAATAGAATCATACA-3'
SiSOD	5'-GGAGCACCAGAAGATGACAACCG-3'	5'-AGTCCAATGATCCCACAGGCAAT-3'
SiSOS1	5'-AATGAGCAAGGAGCACAGCG-3'	5'-TGTTGGAACCCTCCGACTGC-3'
SiLTP	5'-AAGATGACGATGAAGCAGCAG-3'	5'-GTTGGGGCTGTTGATGTAGC-3'
UBQ5	5'-CTCCTTCTTTCTGGTAAACGT-3'	5'-GGTGCTAAGAAGAGGAAGAAT-3'
Siactin	5'-GTGCTTTCCCTCTACGCCAGTG-3'	5'-ACCGCTGAGCACAATGTTACCA-3'
Bait sequences		
DREB	5'-AGCTTACTTGATGATGCCGACCGCTTGC-3'	5'-TCGAGCAAGCGGTCGGCATCATCAAGTA-3'
mDREB	5'-AGCTTACTTGATGATAAAAAACGCTTGC-3'	5'-TCGAGAATAGCTTTTTTTCGATCCGAGCA-3'
